# Correction: Evolutionarily Conserved 5'-3' Exoribonuclease Xrn1 Accumulates at Plasma Membrane-Associated Eisosomes in Post-Diauxic Yeast

**DOI:** 10.1371/journal.pone.0126788

**Published:** 2015-04-17

**Authors:** The PLOS ONE Staff

## Notice of Republication

This article was republished on April 8, 2015, to correct figures that were distorted during the production process. The publisher apologizes for the error. Please download this article again to view the correct version. The originally published, uncorrected article and the republished, corrected article are provided here for reference.

## Supporting Information

S1 File Originally published, uncorrected article. (PDF)

S2 File Republished, corrected article. (PDF)
