# Supplementary figures and images for: Evolutionarily Conserved 5’-3’ Exoribonuclease Xrn1 Accumulates at Plasma Membrane-Associated Eisosomes in Post-Diauxic Yeast
Source: PLoS One. 2015 Mar 26;10(3):e0122770. doi: 10.1371/journal.pone.0122770 (PMC4374687; doi:10.1371/journal.pone.0122770)

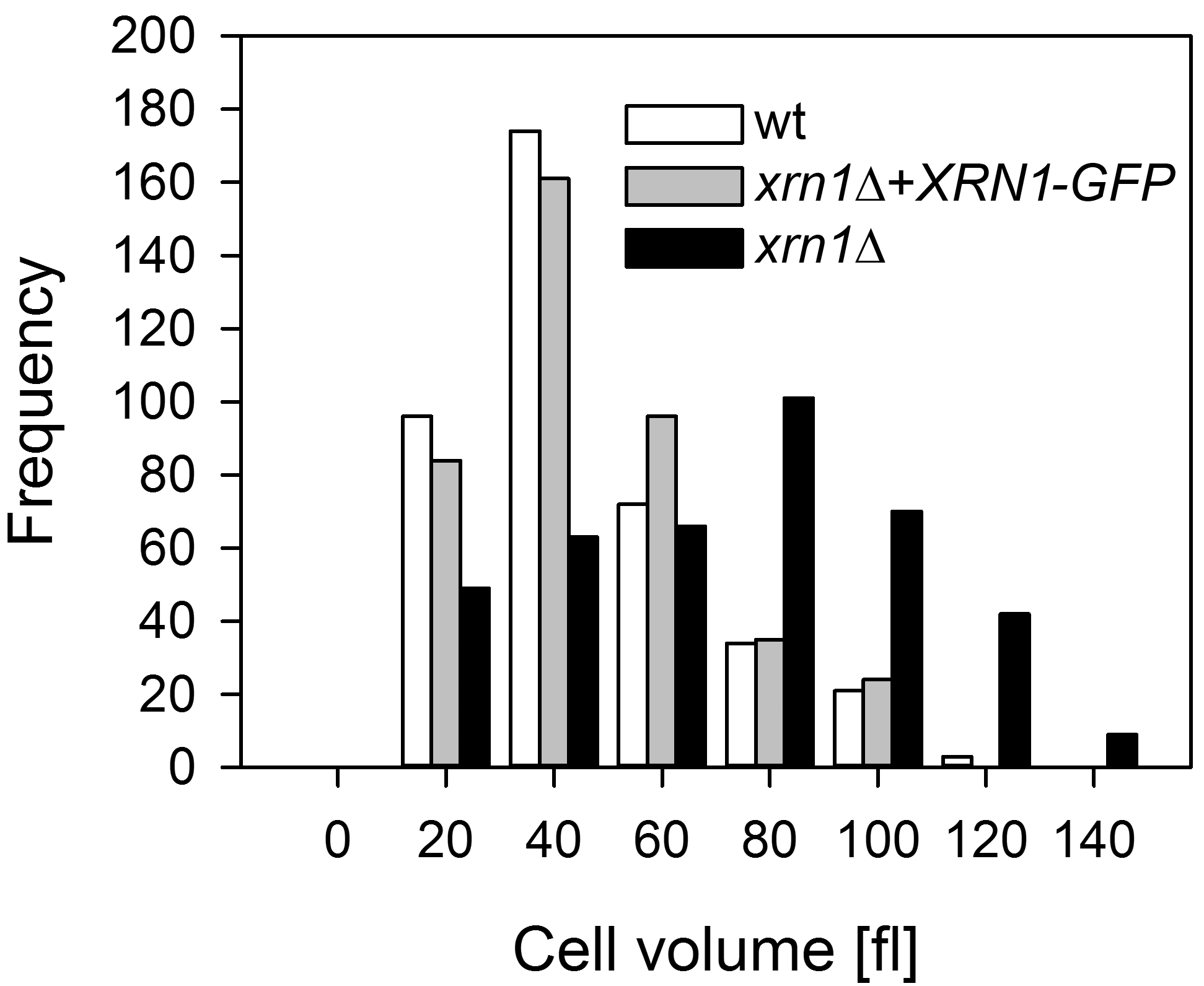

Supplement: S1 Fig — Cell volume was measured in logarithmic cultures of the wild type (empty columns; strain CRY155), xrn1Δ cells (black columns; strain CRY1233) and xrn1Δ cells expressing Xrn1- GFP (grey columns; strain CRY1703) grown on complete synthetic medium supplemented with 2% glucose. For each strain, ≥400 cells were analyzed. Histograms illustrating the cell volume distributions in the respective cultures are presented. Cell volume was assessed as follows: Longest and shortest cell diameters were measured on DIC image in Adobe Photoshop CS4. Shape of each cell was approximated by an elongated (slim) rotational ellipsoid. (TIF) [file pone.0122770.s001.TIF]
