# Supplementary material for: Correction: Evolutionarily Conserved 5'-3' Exoribonuclease Xrn1 Accumulates at Plasma Membrane-Associated Eisosomes in Post-Diauxic Yeast
Source: PLoS One. 2015 Apr 17;10(4):e0126788. doi: 10.1371/journal.pone.0126788 (PMC4401543; doi:10.1371/journal.pone.0126788)
Supplement: S2 File — (PDF) [file pone.0126788.s002.pdf]

PLOS | ONE

RESEARCH ARTICLE

Evolutionarily Conserved 5'-3' Exoribonuclease Xrn1 Accumulates at Plasma Membrane-Associated Eisosomes in Post-Diauxic Yeast

Tomas Grousl[1☯], **Miroslava Opekarová**[2☯], **Vendula Stradalova**[2], **Jiri Hasek**[1]*, **Jan Malinsky**[2]*

1 Institute of Microbiology, Academy of Sciences of the Czech Republic, Prague, Czech Republic, **2** Institute of Experimental Medicine, Academy of Sciences of the Czech Republic, Prague, Czech Republic

☯ These authors contributed equally to this work.
* malinsky@biomed.cas.cz (JM); hasek@biomed.cas.cz (JH)

OPEN ACCESS

Citation: Grousl T, Opekarová M, Stradalova V, Hasek J, Malinsky J (2015) Evolutionarily Conserved 5'-3' Exoribonuclease Xrn1 Accumulates at Plasma Membrane-Associated Eisosomes in Post-Diauxic Yeast. PLoS ONE 10(3): e0122770. doi:10.1371/journal.pone.0122770

Academic Editor: Georg Stoecklin, German Cancer Research Center, GERMANY

Received: October 22, 2014

Accepted: February 18, 2015

Published: March 26, 2015

Copyright: © 2015 Grousl et al. This is an open access article distributed under the terms of the Creative Commons Attribution License, which permits unrestricted use, distribution, and reproduction in any medium, provided the original author and source are credited.

Data Availability Statement: All relevant data are within the paper.

Funding: This work was financially supported by the Czech Science Foundation (projects P302/11/0146 and 15-10641S to M.O., V.S. and J.M.; P304/12/0480 to T.G. and J. H.) and by the institutional grants. The funders had no role in study design, data collection and analysis, decision to publish, or preparation of the manuscript.

Competing Interests: The authors have declared that no competing interests exist.

Abstract

Regulation of gene expression on the level of translation and mRNA turnover is widely conserved evolutionarily. We have found that the main mRNA decay enzyme, exoribonuclease Xrn1, accumulates at the plasma membrane-associated eisosomes after glucose exhaustion in a culture of the yeast *S*. *cerevisiae*. Eisosomal localization of Xrn1 is not achieved in cells lacking the main component of eisosomes, Pil1, or Sur7, the protein accumulating at the membrane compartment of Can1 (MCC) - the eisosome-organized plasma membrane microdomain. In contrast to the conditions of diauxic shift, when Xrn1 accumulates in processing bodies (P-bodies), or acute heat stress, in which these cytosolic accumulations of Xrn1 associate with eIF3a/Rpg1-containing stress granules, Xrn1 is not accompanied by other mRNA-decay machinery components when it accumulates at eisosomes in post-diauxic cells. It is important that Xrn1 is released from eisosomes after addition of fermentable substrate. We suggest that this spatial segregation of Xrn1 from the rest of the mRNA-decay machinery reflects a general regulatory mechanism, in which the key enzyme is kept separate from the rest of mRNA decay factors in resting cells but ready for immediate use when fermentable nutrients emerge and appropriate metabolism reprogramming is required. In particular, the localization of Xrn1 to the eisosome, together with previously published data, accents the relevance of this plasma membrane-associated compartment as a multipotent regulatory site.

Introduction

Regulation of eukaryotic gene expression is a complex process which ensures cell growth, differentiation, adaptation to environmental changes, hormonal stimuli, etc. In addition to direct control of gene transcription levels, post-transcription regulation steps encompassing

especially mRNA turnover and regulation of translation, play essential roles in this process. In the mRNA decay, a majority of the cytosolic mRNAs in eukaryotic cells is degraded by evolutionarily conserved 5'→3' exonuclease XRN1 ([1, 2] and references therein).

In yeast, Xrn1 exonuclease is constitutively expressed at relatively high levels: approx. 11700 molecules per cell [3]. The yeast Xrn1 is engaged in general degradation of mRNA species and also in their quality control (reviewed in [4]). Spatio-temporal segregation of the exonuclease into specialized subcellular compartments represents an important fast-responding and versatile mechanism, through which the cell is able to co-ordinate the mRNA decay process. The "processing bodies" (P-bodies) constitute one of the transient compartments modulating the activity of Xrn1 [5]. P-bodies are ribonucleoprotein (RNP) assemblies in the cytoplasm of eukaryotic cells ranging from yeast to mammals. They are formed in response either to gradually incoming environmental stresses, such as ongoing glucose depletion, or under acute environmental stresses, e.g. heat shock. P-bodies congregate, beside translationally repressed mRNAs and associated factors, also mRNA decay machinery components, such as Xrn1 exonuclease, decapping enzyme subunits and decapping activators [5–8]. Due to this means, it is generally believed that P-bodies take part in regulation of Xrn1-mediated degradation of non-translating mRNAs [5, 9]. In addition, recent data also document an involvement of P-bodies in mRNA quality control, mRNA storage, and translation repression (reviewed in [4, 10]).

Similarly, RNP assemblies called stress granules (SGs) are formed in eukaryotic cells in response to acute environmental stresses. Similarly to P-bodies, SGs contain translationally repressed mRNA molecules [11]. However, the hallmarks of SGs are stalled translation preinitiation complexes and other translation-associated factors [12, 13]. Consequently, the physiological roles of SGs in cell metabolism differ from those of P-bodies. SGs are believed to serve as triage sites, where the further fate of translationally repressed mRNA molecules is determined. As such, SGs represent highly dynamic structures, where mRNA species with associated protein factors shuttle in and out in a coordinated manner (reviewed in [14]). In mammalian cells, it has been shown that mRNAs are in dynamic equilibrium between active translation (polysomes) and SGs. After cessation of stress, mRNAs leave the SGs and either re-enter translation or move to P-bodies for degradation [15, 16]. Similarly, in yeast SGs are believed to serve as transient storage sites for mRNAs on their route back to active translation [17, 18].

Condition-dependent sequestration of specific proteins into steady-state subcellular regions spatially delimits their physiological functionality. In addition to the RNP assemblies denoted above, another well described example of this sequestration of versatile biological functions in defined subcellular region is a compartmentalization of the plasma membrane into lateral microdomains. Areas of specific lipid and protein composition, structure and function have been identified in the plasma membranes of many organisms from bacteria through fungi and plants to mammals [19, 20].

The best described microdomain in the yeast plasma membrane is the Membrane Compartment of arginine permease Can1 (MCC) [21]. MCC possesses characteristic protein and lipid composition (reviewed in [22]) and is organized by cytosolic, membrane-attached complexes called eisosomes [23]. Two major eisosome components, Pil1 and Lsp1, are membrane-sculpting BAR-domain-containing proteins, which assemble in hemi-tubular polymers at the internal face of the plasma membrane and force it to shape into characteristic furrow-like invaginations [24–26]. These membrane furrows are evolutionarily conserved among bacteria, fungi and plants (see [24] for further reference). In addition to Pil1 and Lsp1, about twenty other proteins were found to be localized in the eisosomal complex (for reviews see [22, 27]). Despite extensive research, the biological functions of MCC/eisosome are still not fully understood. The presence of Pkh1/2 kinases, the yeast homologues of 3-phosphoinositide-dependent protein kinase 1 (PDK1), at the eisosome [28], as well as transient MCC/eisosomal

accumulation of other protein factors [29, 30] suggested the involvement of the structure in a signal transduction pathway. Indeed, it has been shown that MCC/eisosome takes part in spingolipid signaling and/or sensing [31], or in cellular response to various types of stress [32, 33].

In this study, we map localization changes of the major *S. cerevisiae* mRNA exoribonuclease Xrn1 in response to evolving limitation in fermentable carbon source. In contrast to acute environmental stresses, e.g. robust heat shock, gradual depletion of nutrients results in re-localization of the Xrn1 enzyme from already-formed P-bodies and its association with MCC/ eisosomes. Our findings suggest a novel, MCC/ eisosome-related, mechanism of mRNA decay regulation.

Materials and Methods

Yeast strains and growth conditions

All *Saccharomyces cerevisiae* strains used in this study are based on BY4741, BY4742 [34] or Sey6210 [35] background and are listed in Table 1. Yeast cultures were cultivated under shaking either in liquid YPD media (1% yeast extract, 2% peptone, 2% glucose) or in liquid SC media (0.17% YNB without amino acids and ammonium sulphate, 0.5% ammonium sulphate, 2% glucose, supplemented with a complete or appropriate mixture of amino acids) at 25°C or 30°C. The corresponding solid media contained 2% (w/v) agar. To select for auxotrophies, the respective amino acid was left out from the dropout mixture. To select for resistance to geneticin, the antibiotic (Sigma-Aldrich, USA) was added to media to the final concentration of 200μg/ml.

Over-night cultures of tested strains were diluted in fresh SC media to an optical density (OD_{600}) = 0.2 and further cultivated at 30°C under shaking. Samples for analysis were taken at

Table 1. Yeast strains used in this study.

BY4741	*MATa his3Δ1 leu2Δ0 met15Δ0 ura3Δ0*	[34]
BY4742	*MATα his3Δ1 leu2Δ0 lys2Δ0 ura3Δ0*	[34]
Sey6210	*MATα leu2-3,112 ura3-52 his3-Δ200 trp1-Δ901 lys2-801 suc2-Δ9*	[35]
CRY155	BY4741; *MATa his3Δ1 leu2Δ0 met15Δ0 ura3Δ0*	[34]
CRY255	Sey6210; *MATα RPG1::mRFP::KanMX4*	[41]
CRY1088	BY4742; *MATα pil1::KanMX4*	Euroscarf
CRY1187	BY4741; *MATa XRN1::GFP::HIS3MX6 + YIp211-PIL1-mRFP::URA3*	This study
CRY1229	BY4741; *MATa XRN1::GFP::HIS3MX6 + YIp128-SUR7-mRFP::LEU2*	This study
CRY1233	BY4741; *MATa xrn1::KanMX4*	Euroscarf
CRY1396	BY4741; *MATa XRN1::GFP::HIS3MX6* + pRP1186 (DCP2-RFP)	This study
CRY1432	CRY1703 x CRY1088; *MATα XRN1::GFP::HIS3MX6 pil1::KanMX4*	This study
CRY1493	BY4742; *MATα sur7::KanMX4*	Euroscarf
CRY1496	CRY1703 x CRY1493; *MATa XRN1::GFP::HIS3MX6 sur7::KanMX4*	This study
CRY1703	BY4741; *MATa XRN1::GFP::HIS3MX6*	[37]
CRY2104	CRY1703 x CRY255; *MATα XRN1::GFP::HIS3MX6 RPG1::mRFP::KanMX4*	This study
CRY2237	BY4742; *MATα lsp1::KanMX4*	Euroscarf
CRY2238	BY4741; *MATa* + pRP1186 (DCP2-RFP)	This study
CRY2241	BY4741; *MATa xrn1::KanMX4* + pRP1186 (DCP2-RFP)	This study
CRY2244	BY4741; *MATa XRN1::GFP::HIS3MX6* + pDhh1-RFP	This study
CRY2245	BY4741; *MATa XRN1::GFP::HIS3MX6* + pRP1574 (Edc3-mCherry)	This study
CRY2278	CRY1703 x CRY2237; *MATa XRN1::GFP::HIS3MX6 lsp1::KanMX4*	This study

doi:10.1371/journal.pone.0122770.t001

indicated time points. To compare growth rates of tested strains on solid media, standard spot test was performed.

To perform the heat shock experiment, over-night culture was diluted in fresh YPD media to an OD_{600} = 0.1–0.2 and further cultivated at 30°C to the exponential phase of an OD_{600} = 0.8. The cells were then collected by brief centrifugation and re-suspended in YPD medium preheated to 46°C and incubated under shaking at the given temperature for additional 10 minutes.

Construction of YIp211-PIL1-mRFP plasmid

The *PIL1* gene was excised from the plasmid *YIp128-PIL1-GFP* [30] as a HindIII-BamHI fragment and ligated into *YIp211-mRFP* plasmid. Before yeast transformation, the plasmid was linearized by digestion with XbaI enzyme.

Construction of strains

CRY1187, CRY1229, CRY2157, CRY2238, CRY2241, CRY2244, CRY2245 and CRY2280 were created by transformation with appropriate plasmids (Table 2). In case of integrative plasmids, correct integration was confirmed by PCR using gene-specific primers. CRY1432, CRY1496, CRY2104, CRY2104 and CRY2278 were made by crossing relevant parental strains, diploid selection, sporulation on solid Fowell media (0.1M KAc, 0.1% yeast extract, 0.05% glucose, 2% agar) and spore dissection using Singer micromanipulator (Singer Instruments, UK). The strains were selected on the basis of tetrad analysis and verified by PCR using gene-specific primers.

Microscopy

The cells cultivated in SC media were concentrated by brief centrifugation and directly mounted on a cover glass and coated with a slice of 1.0%- 1.5% agarose in appropriate media or in 0.17% YNB without amino acids and ammonium sulphate. The cells cultivated in YPD medium were briefly washed in SC medium prior to mounting. All the cells were inspected immediately after the preparation.

Wide field live cell imaging was performed either using Olympus BX60 microscope equipped with a 100x PlanApochromat oil-immersion objective (NA = 1.4), Fluoview cooled CCD camera and an Olympus detection system (HQ-set GFP/EGFP filter block, exc. max. 470, em. max. 525) or Olympus IX-81 inverted microscope equipped with 100x PlanApochromat oil-immersion objective (NA = 1.4), Hammamatsu Orca/ER digital camera and an Olympus CellR detection and analysis system (GFP filter block U-MGFPHQ, exc. max. 488, em. max. 507; RFP filter block U-MWIY2, exc. max. 545–580, em. max. 610). Confocal live cell imaging was performed using Zeiss LSM510 with a 100x PlanApochromat oil-immersion objective (NA = 1.4). Fluorescence signals of GFP and mRFP (excitation 488 nm/Ar laser, and 561 nm/ solid state laser) were detected using the 505–550 nm band-pass, and 580 nm long-pass emission filters, respectively.

Table 2. Plasmids used in this study.

YIp211-PIL1-mRFP	This study
YIp128-SUR7-mRFP	[58]
pRP1186 (DCP2-RFP)	[8]
pDhh1-RFP	unpublished (R.Parker)
pRP1574 (Edc3-mCherry)	[59]

doi:10.1371/journal.pone.0122770.t002

Images were processed using Olympus analySIS or Olympus Xcellence RT software and Adobe Photoshop CS5 software. Pearson's correlation coefficient (r) was used as a measure of statistical correlation between the two fluorescence channels in images of double-labeled cells. Its values range between +1 and -1 and allow distinguishing among positive correlation (co-localization, $r \rightarrow 1$), negative correlation (repulsion, $r \rightarrow -1$) and no correlation (random overlap, $r \approx 0$) of the two signals. The coefficient was calculated using NIH ImageJ software and Co-localization Finder plugin, available at http://rsb.info.nih.gov/ij/plugins/. Presented r value is a mean obtained by the analysis of ~1500 cells from three independent experiments.

Results

Distribution of Xrn1 exoribonuclease within the yeast cell changes in response to glucose exhaustion

Fluorescence tagging of Xrn1, the major mRNA exoribonuclease of *S. cerevisiae*, enabled us to monitor the changes of its localization during cell batch cultivation. Functionality of the Xrn1-GFP fusion protein was tested in two independent experiments. First, decreased growth rate of the *xrn1Δ* cells was rescued to wild-type values by expression of Xrn1-GFP in these cells (Fig. 1A). Second, the absence of Xrn1 induces an increase of the cell size and increased formation of P-bodies [5, 36]. Again, these phenotypes were not observed in Xrn1-GFP expressing strain (S1 Fig. and Fig. 1B-D, respectively).

We inoculated the cells expressing Xrn1-GFP into a synthetic medium (SC) containing a rich carbon source (2% glucose) and followed the development of the culture density. Simultaneously, we monitored the protein distribution over time. Consistently with earlier observations [37, 38], the fluorescence signal of Xrn1-GFP was more or less homogeneously distributed within the cell cytoplasm during the exponential growth of the culture (OD_{600}~0.6; Fig. 2A). When the glucose concentration in the culture became limiting and cells were undergoing diauxic shift, increased formation of processing bodies (P-bodies) in the cytoplasm could be detected. Again, in accordance with the literature data [8, 39], we observed an extensive sequestration of Xrn1-GFP together with other documented P-body markers (Fig. 3A) into these assemblies. Twenty four hours after inoculation, about 90% of the cells in the culture exhibited a fluorescence signal of Xrn1-GFP accumulated in P-bodies (Fig. 2B). P-bodies *per se* subsequently persisted in the cell after the post-diauxic shift to the stationary phase of growth [8, 39, 40]. However, prolonged observation of the cells that obviously accomplished the diauxic shift (post-diauxic cells) revealed a dramatic change in the subcellular distribution of Xrn1. With extended cultivation, the protein accumulated in evenly distributed patches at the cell cortex (Fig. 2C). In contrast, other P-body components, like Dcp2, Dhh1 or Edc3 retained their association with the original RNP assemblies (Fig. 3B). Thirty hours after the inoculation, about 70% of the cells exhibited cortical focal accumulations of Xrn1 (Fig. 2C). This cellular fraction then slowly increased up to more than 80%. The regular cortical pattern of Xrn1 fluorescence could be observed in cells cultivated in the batch culture up to more than 48 hours, when it was gradually dissipated as the cells began to die. Quantification of distinct localization patterns of Xrn1 at the indicated time points in the cell-culture growth is depicted in Fig. 2D. Obviously, the localization of the main fraction of Xrn1 protein successively changed from diffuse cytosolic, through P-bodies-accumulated, to plasma membrane-associated in the exponential, diauxic and post-diauxic cells, respectively. As apparent from Fig. 2A-C, localization patterns of Xrn1 are to a high extent mutually exclusive. In other words, the switch between the successive patterns has to proceed within quite short time windows.

Re-localization of Xrn1 to the cell periphery was observed in post-diauxic cells grown in synthetic medium with 2% glucose independently of the nitrogen source (ammonia ions or

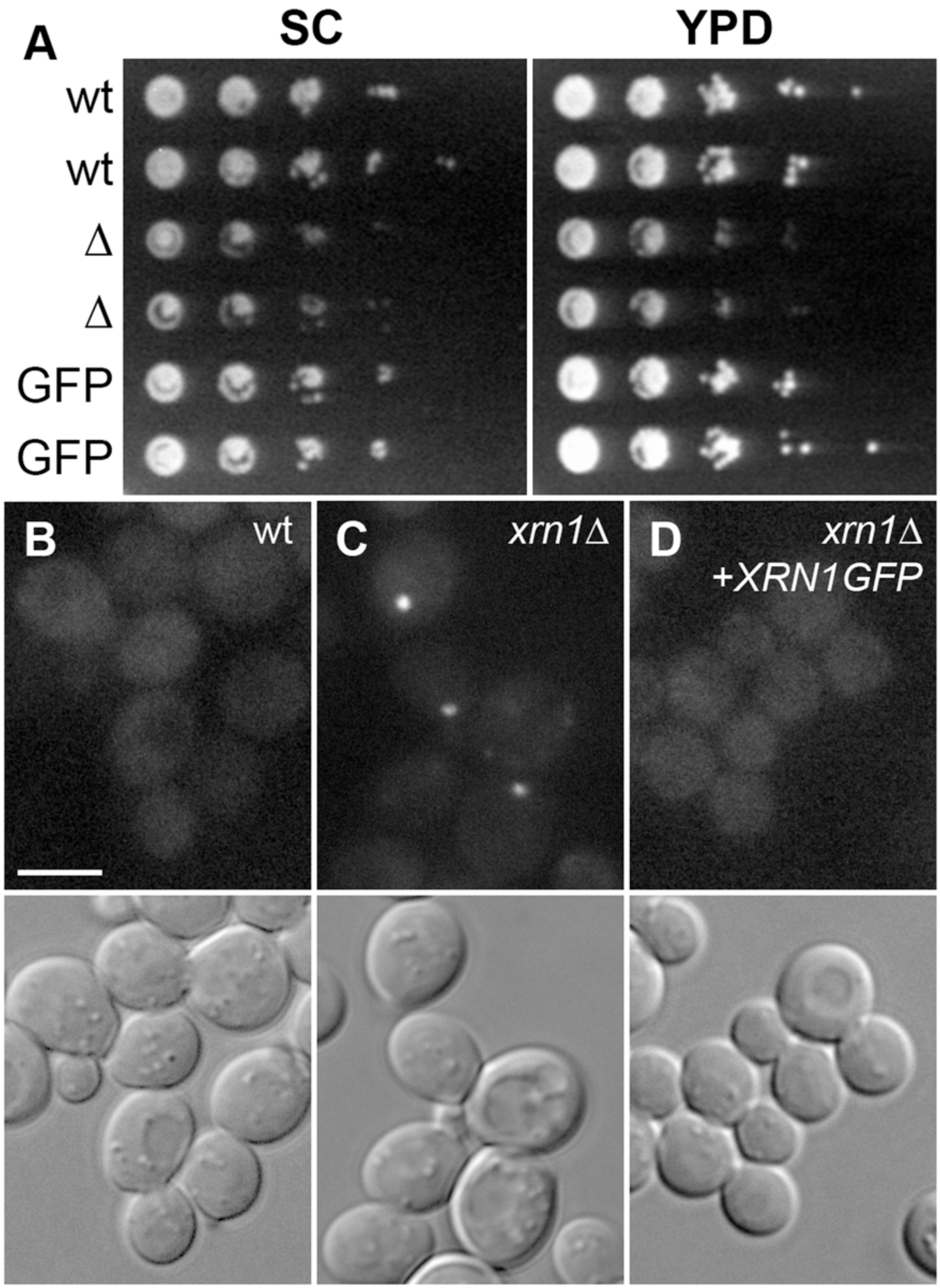

Fig 1. Expression of Xrn1-GFP fusion rescues the *xrn1Δ* phenotype. Standard spot tests comparing the growth rates of the wild type strain (wt in A; strain CRY155), *xrn1Δ* cells (Δ in A; strain CRY1233) and a strain expressing Xrn1-GFP (GFP in A; strain CRY1703) on SC (left panel) and YPD media (right) are shown. Cellular distributions of Dcp2-RFP in the three backgrounds are compared (wild type—B, *xrn1Δ*—C, and a strain expressing Xrn1-GFP—D; strains CRY2238, CRY2241 and CRY1396, respectively). Wide-field fluorescence of Dcp2-RFP and DIC channels are presented in B-D. Bar: 5μm.

doi:10.1371/journal.pone.0122770.g001

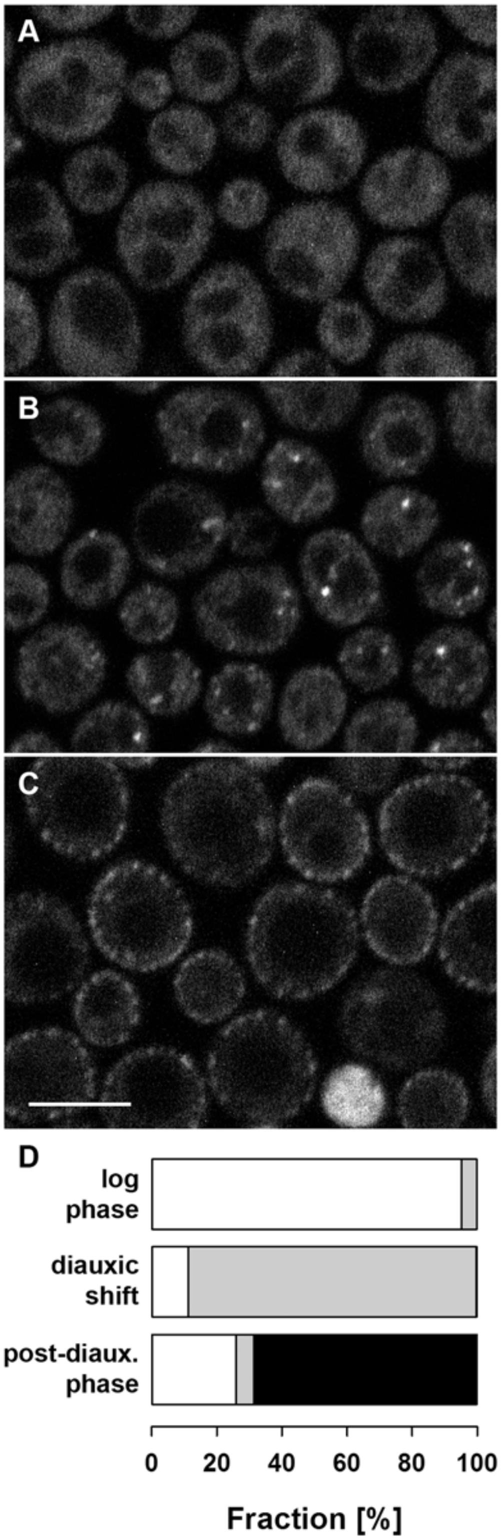

Fig 2. Localization patterns of Xrn1 in three phases of aerobic growth. Cells expressing Xrn1-GFP (strain CRY1703) were observed 3 (A; log phase), 24 (B; diauxic shift), and 30 hours (C; post-diauxic shift) after the inoculation to complete synthetic medium supplemented with 2% glucose. Transversal confocal sections are presented. For each time point documented in A-C, fractions of cells exhibiting Xrn1-GFP fluorescence homogenously distributed in cytosol (white), accumulated in P-bodies (gray) and at evenly distributed cortical patches (black) are depicted (D; n>400 cells were analyzed at each indicated time point). Bar: 5μm.

doi:10.1371/journal.pone.0122770.g002

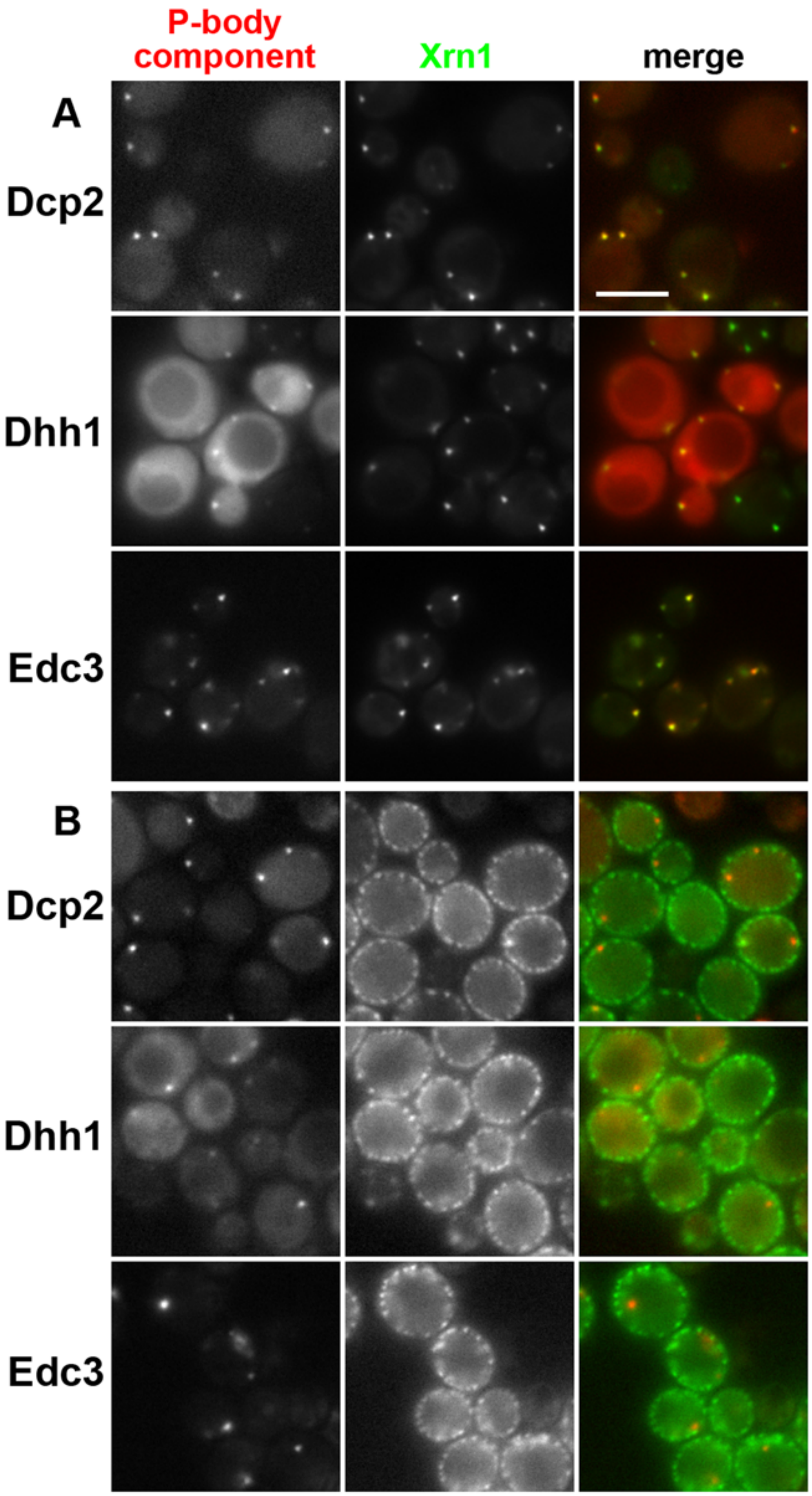

Fig 3. In contrast to other P-body markers, Xrn1 relocalizes to cell cortex in post-diauxic cells. Localization patterns of Xrn1-GFP in diauxic (A) and post-diauxic cells (B) were compared with those of Dcp2-RFP (strain CRY1396), Dhh1-RFP (CRY2244) and Edc3-mCherry (CRY2245). Note that in the diuaxic culture, all the proteins accumulate in P-bodies. In contrast to Xrn1, which accumulates at eisosomes in post-diauxic cells, other P-body marker proteins remain associated with cytoplasmic P-bodies. Wide field images are presented. Bar: 5μm.

doi:10.1371/journal.pone.0122770.g003

proline), and it also occurred in the cells grown on complex medium YPD. Caloric restriction by low glucose concentrations (0.1%; 0.05%) in the starting media resulted in a slower growth rate of the cell culture and lower cell-mass yield in the post-diauxic phase but did not affect the final Xrn1 distribution to a patchy pattern typical for cells grown on 2% of glucose. On the other hand, just a minute fraction (<3%) of cells grown on non-fermentative sources of carbon and energy, like ethanol or glycerol, exhibited Xrn1-GFP accumulation at the cortical patches approximately 48 hours after inoculation, and also later on (further analyzed below).

Xrn1 associates with stress granules after robust heat shock

Assembly of Xrn1-containing P-bodies has been observed not only under conditions of evolving glucose exhaustion during undisturbed yeast culture growth, but also upon acute stresses, for example, following the sudden glucose depletion. Under these conditions, Xrn1 association with the plasma membrane region was, however, not observed [5, 8]. We analyzed the cellular distribution of Xrn1-GFP after a robust heat stress to test whether other types of acute stress could induce the re-localization of Xrn1 to the cell cortex.

As is evident from Fig. 4, Xrn1-GFP did not reach the cell cortex in heat-stressed cells. Instead, together with other P-body constituents, the exoribonuclease accumulated in distinct cytoplasmic foci in these cells. We detected significant overlap of these Xrn1-GFP foci with a marker of stress granules, eIF3a/Rpg1-RFP (Fig. 5). Formation of stress granules in heat-stressed cells has been reported earlier [41]. This observation indicates that robust heat shock triggers an association of Xrn1-containing P-bodies with stress granules.

Cortical accumulation of Xrn1 in post-diauxic cells occurs at MCC/eisosome

The regular cortical pattern of Xrn1 fluorescence in post-diauxic cells bears a resemblance to the distribution of proteins described as components of MCC/eisosomes (reviewed in [22]). We therefore checked the localization of this exoribonuclease in respect to the well-established marker of MCC, Sur7 [42], and the main component of eisosomes, Pil1 [23]. GFP-labeled Xrn1 was expressed in two strains bearing Sur7 or Pil1 C-terminally tagged with red fluorescent protein, respectively. In these strains, cortical accumulations of Xrn1-GFP in post-diauxic cells clearly overlapped with the red fluorescence of both Sur7-mRFP (Fig. 6A-C) and Pil1-mRFP (Fig. 6D-F), indicating that the exonuclease anchors at or in the close vicinity of MCC/eisosome. A minute difference in localization of cortical patches of Sur7 and Pil1 has been described earlier; Pil1-marked eisosomes localized a bit deeper inside the cell as compared to Sur7 protein [24]. Accordingly, cortical patches of Xrn1-GFP fluorescence exactly matched the distribution of Pil1-mRFP eisosomes, while their slight shift towards the cell interior from MCC patches marked by Sur7-GFP fluorescence was observed (compare yellow eisosomes in Fig. 6F with green/red labeling in Fig. 6C).

To demonstrate that the cortical localization of Xrn1 is determined specifically by Sur7 and Pil1, we first expressed Xrn1-GFP in strains bearing deletion of the main eisosome organizer Pil1. In general, Pil1 deletion results in complete redistribution of all the other MCC/eisosome components described so far. MCC proteins like Can1, Sur7 or Nce102 disperse in the whole plasma membrane of *pil1Δ* cells and accumulate in 1–2 big plasma membrane patches per cell, so called eisosome remnants. Soluble eisosomal proteins, like Lsp1, also accumulate in these structures. Aside from eisosome remnants, they do not associate with the plasma membrane [23, 29, 30]. In accordance with this *pil1Δ* phenotype, neither the exoribonuclease Xrn1-GFP in post-diauxic *pil1Δ* cells assembled to its typical pattern at the cell cortex (compare Fig. 6A and 6G). In contrast to that of Pil1, deletion of an integral plasma membrane protein Sur7 has

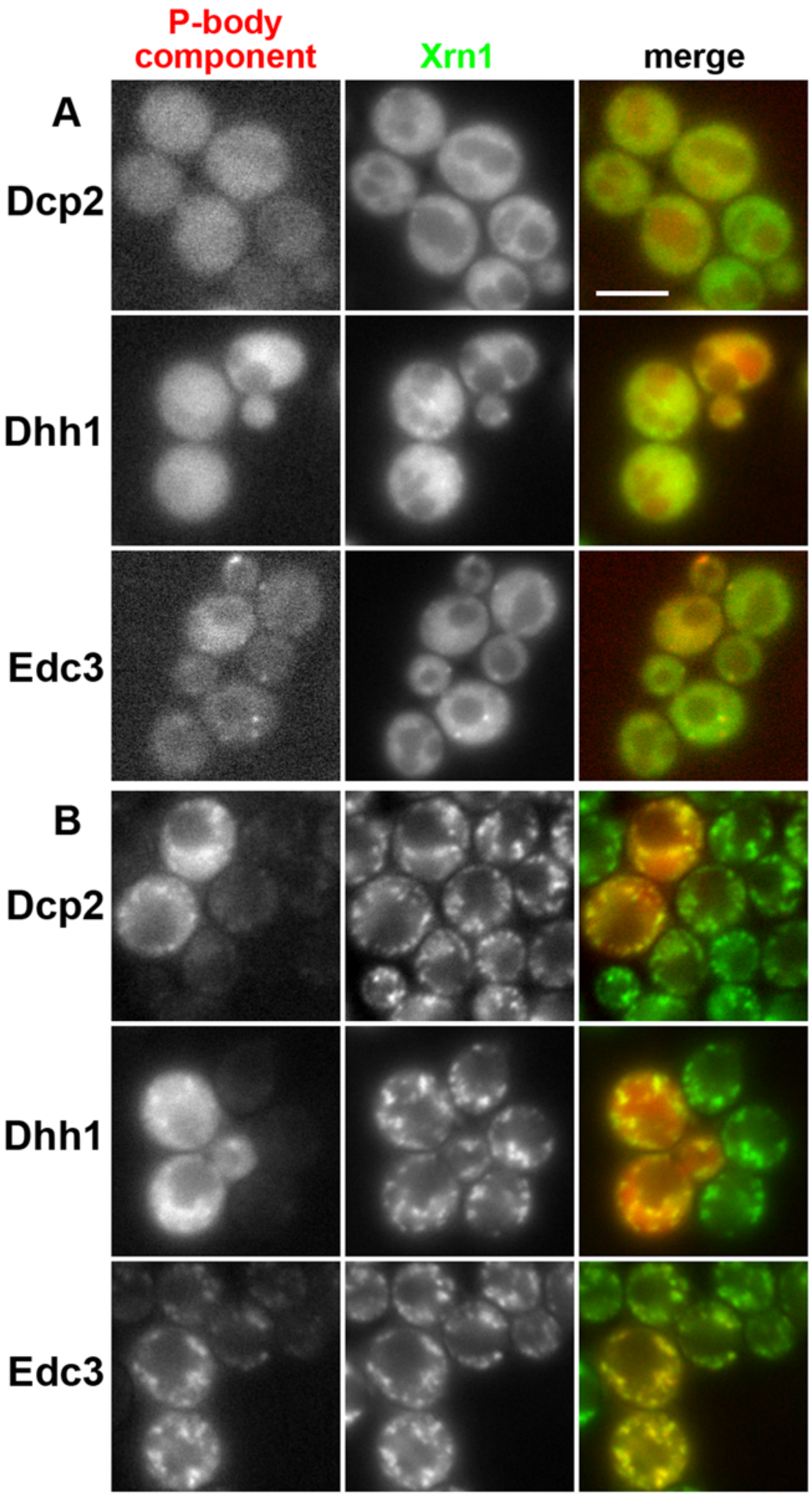

Fig 4. Cellular distributions of Dcp2, Dhh1, Edc3, and Xrn1 overlap under heat stress conditions. Localization patterns of Xrn1-GFP in control (A) and heat-stressed cells (B) were superimposed with the distributions of Dcp2-RFP (strain CRY1396), Dhh1-RFP (CRY2244) and Edc3-mCherry (CRY2245). Note that heat stress induces accumulation of all the proteins in cytosolic granules. Wide field images are presented. Bar: 5μm.

doi:10.1371/journal.pone.0122770.g004

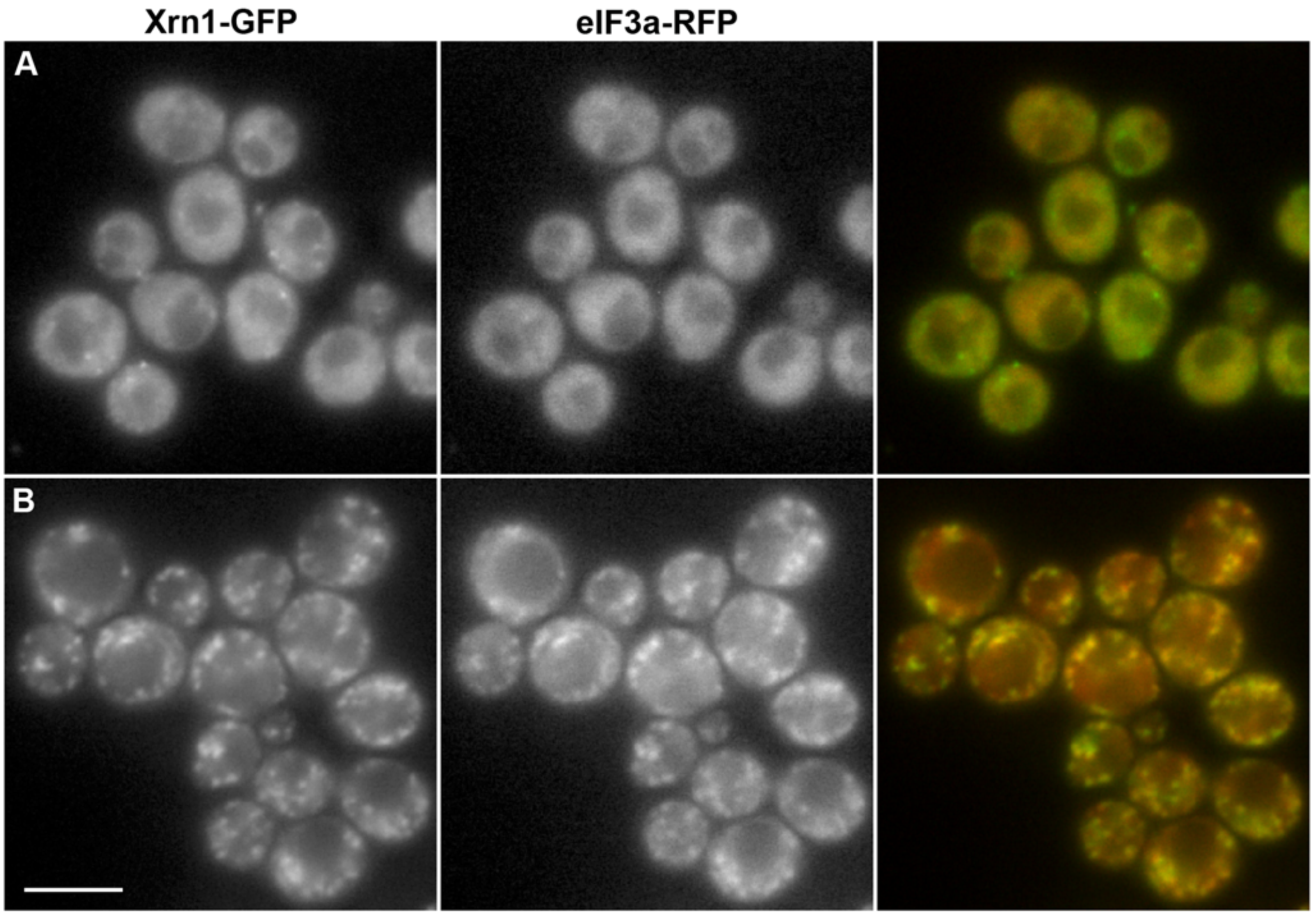

Fig 5. Xrn1 associates with heat-induced stress granules. Localization patterns of Xrn1-GFP and eIF3a-RFP in exponentially growing cells (strain CRY2104) at 30°C (A) and upon heat stress at 46°C (B) were correlated. Note that cytosolic accumulations of Xrn1-GFP significantly overlap with stress granules marked by eIF3a-RFP (Pearson's correlation coefficient r = 0.80 ± 0.02; mean ± SD). Wide field images are presented. Bar: 5μm.

doi:10.1371/journal.pone.0122770.g005

not been observed to disturb the patchy pattern of any other known MCC/eisosome components. Specifically for Can1, Nce102, or Pil1, wild type-like pattern was documented in *sur7Δ* [30]. Nevertheless and interestingly, Sur7 deletion prevented Xrn1-GFP sequestration to otherwise still existing Pil1-organized MCC/eisosomes in post-diauxic cells (Fig. 6H). Based on this observation, we conclude that these two components of MCC/eisosome (Pil1 and Sur7) are necessary for attracting and stabilizing Xrn1 at the cell cortex. Specific involvement of Pil1 and Sur7 in eisosomal localization of Xrn1 is further supported by the fact that deletion of Pil1 analog Lsp1 does not disturb the eisosomal pattern of Xrn1-GFP (Fig. 6I).

Mobilization of fermentative machinery induces re-localization of Xrn1 from MCC/eisosomes back to processing bodies

To test the reversibility of Xrn1 association with MCC/eisosomes, we looked for changes in the distribution of Xrn1-GFP fluorescence after replenishment of glucose in the post-diauxic culture. The cells expressing Xrn1-GFP were inoculated to SC medium with 2% glucose and cultivated for 48 hours when the cortical accumulation of the fluorescent fusion protein was markedly developed. Addition of glucose to this culture induced immediate dissipation of the

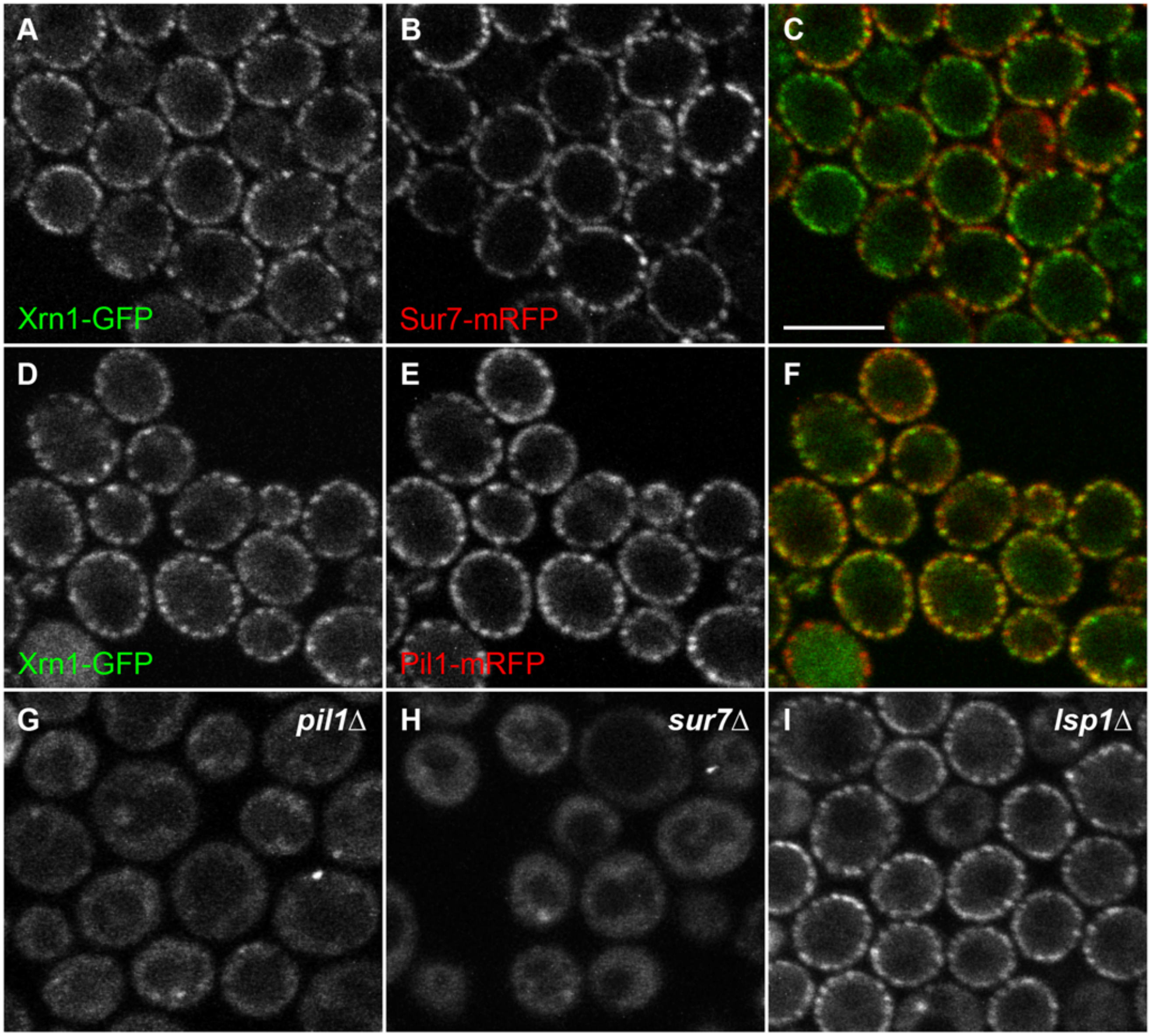

Fig 6. Xrn1 associates with eisosomes in post-diauxic cells. Cellular distribution of Xrn1-GFP (A, D, green in C, F) was compared with those of Sur7-mRFP (B, red in C) and Pil1-mRFP (E, red in F) in wild type post-diauxic cells (strains CRY1229 and CRY1187). Fluorescence patterns of Xrn1-GFP in *pil1Δ* (G; strain CRY1432), *sur7Δ* (H; CRY1496) and *lsp1Δ* cells (I; CRY2278) are also shown. Transversal confocal sections are presented. Bar: 5μm.

doi:10.1371/journal.pone.0122770.g006

MCC/eisosome accumulation of Xrn1-GFP and reversed the protein distribution back to its accumulation in cytosolic P-bodies. A similar effect was observed after addition of a fermentable tri-saccharide, raffinose (for glucose, see Fig. 7A, B). In contrast, addition of non-fermentable carbon sources such as glycerol or ethanol, had no effect on MCC/eisosome Xrn1-GFP localization even after one-hour cultivation of cells with these substrates (for glycerol, see Fig. 7C, D). These results point to direct connection of the eisosomal accumulation of Xrn1 with fermentative metabolism.

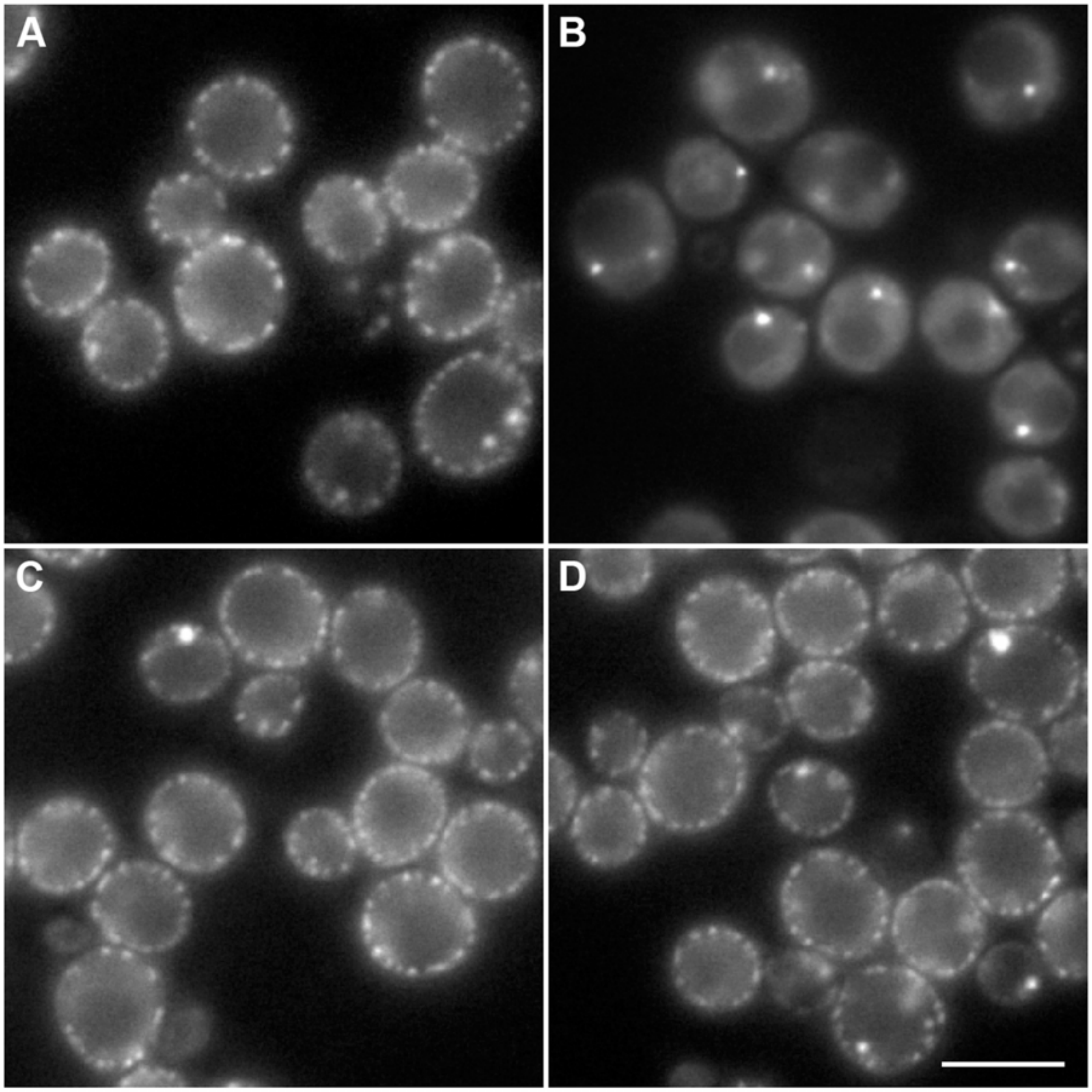

Fig 7. Addition of fermentable carbon source aborts Xrn1 association with MCC/eisosomes. Localization of Xrn1-GFP (strain CRY1703) was monitored in post-diauxic cells (A) and 10 minutes after addition of 2% glucose (B) or 3% glycerol (C) to the culture. While cortical accumulations of Xrn1-GFP rapidly disappeared in glucose-supplied culture and the protein accumulated in P-bodies, addition of glycerol had no effect even after 60 minutes long cultivation (D). Wide field images are presented. Bar: 5μm.

doi:10.1371/journal.pone.0122770.g007

Discussion

Xrn1 is an evolutionarily conserved 5'-3' exoribonuclease involved in the degradation of decapped mRNAs, a terminal step in the mRNA turnover. Together with some other mRNA decay components, Xrn1 localizes into ribonucleoprotein assemblies called P-bodies. P-bodies were shown to participate in repression of translation, mRNA quality control or mRNA storage (reviewed in [43]). Their originally proposed direct involvement in mRNA decay [5] has been questioned in publications by other authors (reviewed in [44]), however, even the most recent studies still anticipate a dual role of P-bodies in both mRNA decay and storage [45]. P-body

formation in cells is observed in response to various environmental stresses. One of these stresses eliciting enhanced P-body formation is progressive glucose depletion from the growth media when the cells approach a diauxic shift [8]. P-bodies then persist through post-diauxic growth to stationary phase [40] (see also Fig. 3). In this study, we report that Xrn1, the inherent P-body marker, does not accumulate in post-diauxic P-bodies.

We show that in post-diauxic cells, Xrn1 leaves the P-body and re-localizes to the cell periphery forming a regular patchy pattern (Fig. 2). By co-localizing these Xrn1 patches with the main eisosome organizer Pil1 and MCC-accumulated integral membrane protein Sur7, and showing that the presence of the two proteins is required for the formation of cortical Xrn1 accumulations (Fig. 6), we demonstrate that Xrn1 accumulates at eisosome under these conditions. While the effect of Pil1 deletion on the Xrn1 cortical localization can be explained by a complete dissipation of the plasma membrane compartments, the effect of Sur7 removal can be only speculated on at the moment. No marked direct consequences of Sur7 deletions were observed in *S. cerevisie*, while analysis of a *C. albicans sur7* mutant revealed a wide range of phenotypes. The most striking among them was an abnormal cell wall synthesis, including long projections of cell wall into the cytoplasm [46]. The participation of Sur7 in organizing the Xrn1 plasma membrane association in postdiauxic cells may point to novel function of Sur7 in *S. cerevisiae*.

The findings above prompt a question about the possible link of the plasma membrane compartment, MCC/eisosome, to mRNA metabolism. Since the introduction of eisosomes to the scientific public, their function has been intensely studied. While the originally proposed eisosome function as a platform for endocytosis initiation [23] has been ruled out [30, 47], further investigations have revealed their involvement in sensing, signaling and stress response (reviewed in [20]). Core eisosome components Pil1 and Lsp1 were found in a complex with functionally redundant homologues of mammalian phosphoinositide-dependent protein kinase-1, Pkh1/2 [48]. Pil1 and Lsp1 were suggested to regulate the activities of Pkh1/2 and, consequently, their downstream regulation pathways controlling cell growth and survival [49]. Finally, cortical accumulations of Pkh1/2 were co-localized with eisosomes [28] and activity of Pkh kinases was shown to be required for eisosome stability [28, 50].

Direct linking of Pkh1/2 and their downstream effector Pkc1 to mRNA decay in yeast was demonstrated by Luo et al. (2011) [51]. Using synthetic genetic array analysis with *pkh1*^{ts}*pkh2* as a query strain, they identified a synthetic growth defect of two strains with deletions of important genes involved in mRNA decay: *DHH1* gene coding for a DEXD/H-box helicase that stimulates mRNA decapping, represses translation and functions in P-body formation, and *XRN1* gene, coding for the main yeast 5'-3' exoribonuclease. Examining the synthetic slow-growing strain bearing *DHH1* deletion with *pkh1*^{ts}*pkh2* the authors could show that Pkh1/2 kinase exerts an effect both on P-body formation and, *via* mRNA deadenylation, on mRNA decay. The other synthetic slow growth interaction with *XRN1* was not explored, but it can be expected on basis of the above study that Pkh1/2 also controls the Xrn1 activity. Remarkably, this effect could be observed only in cells grown on nutrient-poor media, i.e. in conditions comparable to the post-diauxic culture, in which we document accumulation of Xrn1 at Pkh1/2-containing eisosomes (Fig. 6).

In contrast to a progressive stress caused by evolving glucose depletion, neither acute glucose deprivation stress [5, 8] nor acute heat stress (Figs. 4 and 5) resulted in re-localization of Xrn1 to the cell cortex. Instead, we found that local cytosolic accumulations of Xrn1-GFP fluorescence observed in heat-stressed cells overlapped with another type of RNP assemblies, stress granules (Fig. 5), the formation of which in response to a robust heat shock had been described earlier [41]. Therefore, it is legitimate to assume that the association with SGs reflects the local distribution of Xrn1 exoribonuclease activity. Since mammalian XRN1 was also reported to

associate with SGs [16], it seems that at least this localization of the 5'-3' exoribonuclease is widely conserved. In addition, earlier findings indicated that even accumulations of other P-body components, Dcp2 and Dhh1, overlap with SGs upon robust heat stress [41] (see also Fig. 4). This suggests that the formation of P-bodies under the conditions of the acute stress occurs in association with SGs.

Several indications lead us to a conclusion that the cortical accumulation of Xrn1 also has an important physiological significance: i) it occurs under defined metabolic conditions (Fig. 2). Notably, progressive carbon source depletion is probably one of the most frequent challenges in cell life in general; ii) it occurs at the eisosome (Fig. 6), a place where the signaling cascade of Pkh kinases begins. This localization is therefore favorable for fast response to sensing events; iii) it spatially separates Xrn1 from the rest of the mRNA decay machinery. We found that Dcp2, Dhh1 or Edc3 remain accumulated in P-body in post-diauxic cells (Fig. 3B).

Relocation of Xrn1 from cytoplasm to P-bodies in diauxic cells occurs as a consequence of the fermentation-to-respiration shift, when the cell has to dramatically re-program the transcription. An enormous amount of mRNAs coding the proteins necessary for glucose fermentation is decomposed at this stage. Some mRNA molecules remain accumulated in P-bodies after the task is accomplished. Importantly, a fraction of the mRNA species accumulated in the P-bodies can be returned to translation [52], even during the stationary phase [53]. In other words the P-body seems to serve as a place for retention, not degradation of mRNA in nutrition-limited cells. It is worth noting in this context that P-bodies have indeed been described as being important for the long-term survival of yeast cells in the stationary phase of growth [39, 40]. Molecular details of the P-body-related mechanism conferring viability of quiescent cells, however, remain unknown. Together with these data, the obvious absence of Xrn1 in the P-bodies of post-diauxic cells (Fig. 3B) suggests that otherwise seemingly intact P-bodies adopt a distinct metabolic function in these cells. The reasons for retention of specific mRNAs are no longer valid with re-appearance of fermentable nutrients. Accordingly, we do, in fact, observe immediate re-location of Xrn1 back to cytosol and P-bodies in response to a fermentable carbon source supply (Fig. 7).

Based on these findings we propose a model of the mRNA decay regulation, in which Xrn1 exoribonuclease activity is regulated by physical separation of the enzyme from the rest of mRNA decay machinery. This consideration is also supported by the study of Aragon et al. [54] documenting that P-bodies in the stationary growth phase contain intact extraction-resistant mRNAs, which can be specifically released to the cytoplasm in response to various types of stress. The stress-induced increase in the abundance of the mRNAs was estimated by the microarray test where only polyadenylated mRNAs can be detected. The whole set of polyadenylated mRNAs preserved in P-bodies could be released by protease treatment, confirming the mRNA protection in a protein complex. In contrast, the same treatment performed with exponential cells did not result in mRNA extraction responsive to the microarray test. We interpret the release of intact mRNAs from stationary-cell lysates by protease treatment as an indication of a lack of complete decay machinery in P-bodies. As we show in these cells, the Xrn1 exoribonuclease is separated from Dcp2, Dhh1 and Edc3, enzymes ensuring key steps in mRNA decay, and localizes to plasma membrane-associated eisosomes (Fig. 3). It is noteworthy here that the eisosomal accumulation of Xrn1 in post-diauxic yeast is not the first observation of a direct participation of the plasma membrane in a spatio-temporal regulation of the mRNA turnover. It was documented that the membrane binding of RNase E stabilizes the protein structure and increases an affinity to its RNA substrate in *E. coli* [55, 56]. The final proof of regulation of Xrn1 activity at the eisosome still needs to be performed, however.

Along with the versatile formerly described functional involvements of eisosome, temporal accumulation of Xrn1 confirms that this plasma membrane-associated compartment plays the

role of a signaling hub, distributing the sensed environmental stimuli through Pkh1/2 kinases to their targets. These targets include stress response [32, 33], lipid homeostasis [31, 57] and mRNA decay pathways ([51] and this study). Notably, the eisosome itself, Pkh kinases, and their targets including Xrn1 exoribonuclease are widely conserved evolutionarily, which supports a general relevance of the proposed model.

Supporting Information

S1 Fig. Expression of Xrn1-GFP fusion rescues the *xrn1Δ* cell-size defect. Cell volume was measured in logarithmic cultures of the wild type (empty columns; strain CRY155), *xrn1Δ* cells (black columns; strain CRY1233) and *xrn1Δ* cells expressing Xrn1- GFP (grey columns; strain CRY1703) grown on complete synthetic medium supplemented with 2% glucose. For each strain, ≥400 cells were analyzed. Histograms illustrating the cell volume distributions in the respective cultures are presented. Cell volume was assessed as follows: Longest and shortest cell diameters were measured on DIC image in Adobe Photoshop CS4. Shape of each cell was approximated by an elongated (slim) rotational ellipsoid.
(TIF)

Acknowledgments

The authors are grateful to Widmar Tanner (University of Regensburg, Germany) for motivating discussions and critical reading of the manuscript. The authors would like to thanks also to Roy Parker (University of Arizona, Tuscon, AZ) for providing pRP1186, pDhh1-RFP and pRP1574 plasmids.

Author Contributions

Conceived and designed the experiments: TG MO. Performed the experiments: TG MO JM VS. Analyzed the data: TG MO JM VS. Wrote the paper: TG MO JM JH.

References

1. Haimovich G, Medina DA, Causse SZ, Garber M, Millán-Zambrano G, Barkai O, et al. Gene expression is circular: factors for mRNA degradation also foster mRNA synthesis. Cell. 2013; 153(5): 1000–11. doi: 10.1016/j.cell.2013.05.012 PMID: 23706738
2. Nagarajan VK, Jones CI, Newbury SF, Green PJ. XRN 5'—>3' exoribonucleases: structure, mechanisms and functions. Biochimica et biophysica acta. 2013; 1829(6–7): 590–603. doi: 10.1016/j.bbagrm.2013.10.003 PMID: 24185200
3. Ghaemmaghami S, Huh WK, Bower K, Howson RW, Belle A, Dephoure N, et al. Global analysis of protein expression in yeast. Nature. 2003; 425(6959): 737–41. PMID: 14562106
4. Parker R. RNA degradation in *Saccharomyces cerevisae*. Genetics. 2012; 191(3): 671–702. doi: 10.1534/genetics.111.137265 PMID: 22785621
5. Sheth U, Parker R. Decapping and decay of messenger RNA occur in cytoplasmic processing bodies. Science. 2003; 300(5620): 805–808. PMID: 12730603
6. Bashkirov VI, Scherthan H, Solinger JA, Buerstedde JM, Heyer WD. A mouse cytoplasmic exoribonuclease (mXRN1p) with preference for G4 tetraplex substrates. J Cell Biol. 1997; 136(4): 761–73. PMID: 9049243
7. Ingelfinger D, Arndt-Jovin DJ, Lührmann R, Achsel T. The human LSm1-7 proteins colocalize with the mRNA-degrading enzymes Dcp1/2 and Xrnl in distinct cytoplasmic foci. RNA. 2002; 8(12): 1489–501. PMID: 12515382
8. Teixeira D, Sheth U, Valencia-Sanchez MA, Brengues M, Parker R. Processing bodies require RNA for assembly and contain nontranslating mRNAs. RNA. 2005; 11(4): 371–82. PMID: 15703442
9. Parker R, Sheth U, P bodies and the control of mRNA translation and degradation. Mol Cell. 2007; 25 (5): 635–46. PMID: 17349952

10. Balagopal V, Parker R. Polysomes, P bodies and stress granules: states and fates of eukaryotic mRNAs. Curr Opin Cell Biol. 2009; 21(3): 403–8. doi: 10.1016/j.ceb.2009.03.005 PMID: 19394210
11. Kedersha NL, Gupta M, Li W, Miller I, Anderson P. RNA-binding proteins TIA-1 and TIAR link the phosphorylation of eIF-2 alpha to the assembly of mammalian stress granules. J Cell Biol. 1999; 147(7): 1431–42. PMID: 10613902
12. Kedersha N, Chen S, Gilks N, Li W, Miller IJ, Stahl J, et al. Evidence that ternary complex (eIF2-GTP-tRNA(i)(Met))-deficient preinitiation complexes are core constituents of mammalian stress granules. Mol Biol Cell. 2002; 13(1): 195–210. PMID: 11809833
13. Kimball SR, Horetsky RL, Ron D, Jefferson LS, Harding HP. Mammalian stress granules represent sites of accumulation of stalled translation initiation complexes. Am J Physiol Cell Physiol. 2003; 284 (2): C273–84. PMID: 12388085
14. Anderson P, Kedersha N. Stress granules: the Tao of RNA triage. Trends Biochem Sci. 2008; 33(3): 141–50. doi: 10.1016/j.tibs.2007.12.003 PMID: 18291657
15. Kedersha N, Cho MR, Li W, Yacono PW, Chen S, Gilks N, et al. Dynamic shuttling of TIA-1 accompanies the recruitment of mRNA to mammalian stress granules. J Cell Biol. 2000; 151(6): 1257–68. PMID: 11121440
16. Kedersha N, Stoecklin G, Ayodele M, Yacono P, Lykke-Andersen J, Fritzler MJ, et al. Stress granules and processing bodies are dynamically linked sites of mRNP remodeling. J Cell Biol. 2005; 169(6): 871–84. PMID: 15967811
17. Hoyle NP, Castelli LM, Campbell SG, Holmes LE, Ashe MP. Stress-dependent relocalization of translationally primed mRNPs to cytoplasmic granules that are kinetically and spatially distinct from P-bodies. J Cell Biol. 2007; 179(1): 65–74. PMID: 17908917
18. Buchan JR, Yoon JH, Parker R. Stress-specific composition, assembly and kinetics of stress granules in *Saccharomyces cerevisiae*. J Cell Sci. 2011; 124(Pt 2): 228–39. doi: 10.1242/jcs.078444 PMID: 21172806
19. Simons K, Gerl MJ. Revitalizing membrane rafts: new tools and insights. Nature reviews. Mol Cell Biol. 2010; 11(10): 688–99. doi: 10.1038/nrm2977 PMID: 20861879
20. Malinsky J, Opekarová M, Grossmann G, Tanner W. Membrane microdomains, rafts, and detergent-resistant membranes in plants and fungi. Annu Review Plant Biol. 2013; 64: 501–29. doi: 10.1146/annurev-arplant-050312-120103 PMID: 23638827
21. Malínská K, Malínský J, Opekarová M, Tanner W. Visualization of protein compartmentation within the plasma membrane of living yeast cells. Mol Biol Cell. 2003; 14(11): 4427–36. PMID: 14551254
22. Malinsky J, Opekarova M, Tanner W. The lateral compartmentation of the yeast plasma membrane. Yeast. 2010; 27(8): 473–8. doi: 10.1002/yea.1772 PMID: 20641012
23. Walther TC, Brickner JH, Aguilar PS, Bernales S, Pantoja C, Walter P. Eisosomes mark static sites of endocytosis. Nature. 2006; 439(7079): 998–1003. PMID: 16496001
24. Strádalová V, Stahlschmidt W, Grossmann G, Blazíková M, Rachel R, Tanner W, et al. Furrow-like invaginations of the yeast plasma membrane correspond to membrane compartment of Can1. J Cell Sci. 2009; 122(Pt 16): 2887–94. doi: 10.1242/jcs.051227 PMID: 19638406
25. Olivera-Couto A, Graña M, Harispe L, Aguilar PS. The eisosome core is composed of BAR domain proteins. Mol Biol Cell. 2011; 22(13): 2360–72. doi: 10.1091/mbc.E10-12-1021 PMID: 21593205
26. Karotki L, Huiskonen JT, Stefan CJ, Ziółkowska NE, Roth R, Surma MA, et al. Eisosome proteins assemble into a membrane scaffold. J Cell Biol. 2011; 195(5): 889–902. doi: 10.1083/jcb.201104040 PMID: 22123866
27. Douglas LM, Konopka JB, Fungal Membrane Organization: The Eisosome Concept. Annu Rev Microbiol. 2014; 68: 377–93. doi: 10.1146/annurev-micro-091313-103507 PMID: 25002088
28. Walther TC, Aguilar PS, Fröhlich F, Chu F, Moreira K, Burlingame AL et al. Pkh-kinases control eisosome assembly and organization. EMBO J. 2007; 26(24): 4946–55. PMID: 18034155
29. Grossmann G, Opekarová M, Malinsky J, Weig-Meckl I, Tanner W. Membrane potential governs lateral segregation of plasma membrane proteins and lipids in yeast. EMBO J. 2007; 26(1): 1–8. PMID: 17170709
30. Grossmann G, Malinsky J, Stahlschmidt W, Loibl M, Weig-Meckl I, Frommer WB et al. Plasma membrane microdomains regulate turnover of transport proteins in yeast. J Cell Biol. 2008; 183(6): 1075–1088. doi: 10.1083/jcb.200806035 PMID: 19064668
31. Fröhlich F, Moreira K, Aguilar PS, Hubner NC, Mann M, Walter P, et al. A genome-wide screen for genes affecting eisosomes reveals Nce102 function in sphingolipid signaling. J Cell Biol. 2009; 185(7): 1227–42. doi: 10.1083/jcb.200811081 PMID: 19564405

32. Dupont S, Beney L, Ritt JF, Lherminier J, Gervais P. Lateral reorganization of plasma membrane is involved in the yeast resistance to severe dehydration. BBA. 2010; 1798(5): 975–85. doi: 10.1016/j.bbamem.2010.01.015 PMID: 20116363

33. Berchtold D, Piccolis M, Chiaruttini N, Riezman I, Riezman H, Roux A, et al. Plasma membrane stress induces relocalization of Slm proteins and activation of TORC2 to promote sphingolipid synthesis. Nat Cell Biol. 2012; 14(5): 542–7. doi: 10.1038/ncb2480 PMID: 22504275

34. Brachmann CB, Davies A, Cost GJ, Caputo E, Li J, Hieter P, et al. Designer deletion strains derived from *Saccharomyces cerevisiae S288C*: a useful set of strains and plasmids for PCR-mediated gene disruption and other applications. Yeast. 1998; 14(2): 115–32. PMID: 9483801

35. Robinson JS, Klionsky DJ, Banta LM, Emr SD. Protein sorting in Saccharomyces cerevisiae: isolation of mutants defective in the delivery and processing of multiple vacuolar hydrolases. Mol Cell Biol. 1988; 8(11): 4936–48. PMID: 3062374

36. Jorgensen P, Nishikawa JL, Breitkreutz BJ, Tyers M. Systematic identification of pathways that couple cell growth and division in yeast. Science. 2002; 297(5580): 395–400. PMID: 12089449

37. Huh WK, Falvo JV, Gerke LC, Carroll AS, Howson RW, Weissman JS et al. Global analysis of protein localization in budding yeast. Nature. 2003; 425(6959): 686–91. PMID: 14562095

38. Mitchell SF, Jain S, She M, Parker R. Global analysis of yeast mRNPs. Nat Struct Mol Biol. 2013; 20(1): 127–33. doi: 10.1038/nsmb.2468 PMID: 23222640

39. Ramachandran V, Shah KH, Herman PK. The cAMP-dependent protein kinase signaling pathway is a key regulator of P body foci formation. Mol Cell. 2011; 43(6): 973–81. doi: 10.1016/j.molcel.2011.06.032 PMID: 21925385

40. Shah KH, Zhang B, Ramachandran V, Herman PK. Processing Body and Stress Granule Assembly Occur by Independent and Differentially Regulated Pathways in *Saccharomyces cerevisiae*. Genetics. 2013; 193(1): 109–23. doi: 10.1534/genetics.112.146993 PMID: 23105015

41. Grousl T, Ivanov P, Frýdlová I, Vasicová P, Janda F, Vojtová J, et al. Robust heat shock induces eIF2 alpha-phosphorylation-independent assembly of stress granules containing eIF3 and 40S ribosomal subunits in budding yeast, *Saccharomyces cerevisiae*. J Cell Sci. 2009; 122(12): 2078–2088. doi: 10.1242/jcs.045104 PMID: 19470581

42. Malinska K, Malinsky J, Opekarova M, Tanner W. Distribution of Can1p into stable domains reflects lateral protein segregation within the plasma membrane of living *S*. *cerevisiae* cells. J Cell Sci. 2004; 117 (Pt 25): 6031–41. PMID: 15536122

43. Eulalio A, Behm-Ansmant I, Izaurralde E. P bodies: at the crossroads of post-transcriptional pathways. Nature reviews. Mol Cell Biol. 2007; 8(1): 9–22.

44. Balagopal V, Fluch L, Nissan T. Ways and means of eukaryotic mRNA decay. BBA. 2012; 1819(6): 593–603. doi: 10.1016/j.bbagrm.2012.01.001 PMID: 22266130

45. Aizer A, Kalo A, Kafri P, Shraga A, Ben-Yishay R, Jacob A, et al. Quantifying mRNA targeting to P bodies in living human cells reveals a dual role in mRNA decay and storage. J Cell Sci. 2014; 127(20): 4443–56.

46. Alvarez FJ, Douglas LM, Rosebrock A, Konopka JB. The Sur7 protein regulates plasma membrane organization and prevents intracellular cell wall growth in *Candida albicans*. Mol Biol Cell. 2008; 19(12): 5214–25. doi: 10.1091/mbc.E08-05-0479 PMID: 18799621

47. Brach T, Specht T, Kaksonen M, Reassessment of the role of plasma membrane domains in the regulation of vesicular traffic in yeast. J Cell Sci. 2011; 124(3): 328–37.

48. Roelants FM, Torrance PD, Bezman N, Thorner J. Pkh1 and Pkh2 differentially phosphorylate and activate Ypk1 and Ykr2 and define protein kinase modules required for maintenance of cell wall integrity. Mol Biol Cell. 2002; 13(9): 3005–28. PMID: 12221112

49. Zhang X, Lester RL, Dickson RC. Pil1p and Lsp1p negatively regulate the 3-phosphoinositide-dependent protein kinase-like kinase Pkh1p and downstream signaling pathways Pkc1p and Ypk1p. J Biol Chem. 2004; 279(21): 22030–8. PMID: 15016821

50. Luo G, Gruhler A, Liu Y, Jensen ON, Dickson RC. The sphingolipid long-chain base-Pkh1/2-Ypk1/2 signaling pathway regulates eisosome assembly and turnover. J Biol Chem. 2008; 283(16): 10433–44. doi: 10.1074/jbc.M709972200 PMID: 18296441

51. Luo G, Costanzo M, Boone C, Dickson RC. Nutrients and the Pkh1/2 and Pkc1 protein kinases control mRNA decay and P-body assembly in yeast. J Biol Chem. 2011; 286(11): 8759–70. doi: 10.1074/jbc.M110.196030 PMID: 21163942

52. Arribere JA, Doudna JA, Gilbert WV. Reconsidering movement of eukaryotic mRNAs between polysomes and P bodies. Mol Cell. 2011; 44(5): 745–58. doi: 10.1016/j.molcel.2011.09.019 PMID: 22152478

53. Brengues M, Teixeira D, Parker R. Movement of eukaryotic mRNAs between polysomes and cytoplasmic processing bodies. Science. 2005; 310(5747): 486–9. PMID: 16141371

54. Aragon AD, Quiñones GA, Thomas EV, Roy S, Werner-Washburne M. Release of extraction-resistant mRNA in stationary phase *Saccharomyces cerevisiae* produces a massive increase in transcript abundance in response to stress. Genome Biol. 2006; 7(2): R9. PMID: 16507144

55. Murashko ON, Kaberdin VR, Lin-Chao S. Membrane binding of *Escherichia coli* RNase E catalytic domain stabilizes protein structure and increases RNA substrate affinity. Proc Natl Acad Sci U S A. 2012; 109(18): 7019–24. doi: 10.1073/pnas.1120181109 PMID: 22509045

56. Mackie GA. RNase E: at the interface of bacterial RNA processing and decay. Nature reviews. Microbiology. 2013; 11(1): 45–57. doi: 10.1038/nrmicro2930 PMID: 23241849

57. Fröhlich F, Christiano R, Olson DK, Alcazar-Roman A, DeCamilli P, Walther TC. A role for eisosomes in maintenance of plasma membrane phosphoinositide levels. Mol Biol Cell. 2014; 25(18): 2797–806. doi: 10.1091/mbc.E13-11-0639 PMID: 25057013

58. Stradalova V, Blazikova M, Grossmann G, Opekarová M, Tanner W, Malinsky J. Distribution of cortical endoplasmic reticulum determines positioning of endocytic events in yeast plasma membrane. PLOS ONE. 2012; 7(4): e35132. doi: 10.1371/journal.pone.0035132 PMID: 22496901

59. Buchan JR, Muhlrad D, Parker R. P bodies promote stress granule assembly in *Saccharomyces cerevisiae*. J Cell Biol. 2008; 183(3): 441–55. doi: 10.1083/jcb.200807043 PMID: 18981231
